# Statistical recommendations for count, binary, and ordinal data in rare disease cross-over trials

**DOI:** 10.1186/s13023-023-02990-1

**Published:** 2023-12-19

**Authors:** Martin Geroldinger, Johan Verbeeck, Andrew C. Hooker, Konstantin E. Thiel, Geert Molenberghs, Joakim Nyberg, Johann Bauer, Martin Laimer, Verena Wally, Arne C. Bathke, Georg Zimmermann

**Affiliations:** 1 Team Biostatistics and Big Medical Data, IDA Lab Salzburg, Paracelsus Medical University, Strubergasse 21, Salzburg, 5020 Austria; 2 Department of Neurology, Christian Doppler Medical Centre, Full Member of European Reference Network on Rare and Complex Epilepsies EpiCARE, Paracelsus Medical University, Ignaz-Harrer Straße 79, Salzburg, 5020 Austria; 3 I-BioStat, Hasselt University, Martelarenlaan 42, 3500 Hasselt, Belgium; 4 I-BioStat, KU Leuven, Kapucijnenvoer 35, 3000 Leuven, Belgium; 5 Department of Pharmacy, Uppsala University, 751 24 Uppsala, Sweden; 6 Department of Dermatology and Allergology, Paracelsus Medical University, Salzburg, 5020 Austria; 7 EB House Austria, Research Program for Molecular Therapy of Genodermatoses, Department of Dermatology and Allergology, University Hospital of the Paracelsus Medical University Salzburg, Salzburg, 5020 Austria; 8 Intelligent Data Analytics (IDA) Lab Salzburg, Department of Artificial Intelligence and Human Interfaces, University of Salzburg, Salzburg, 5020 Austria

**Keywords:** Cross-over, Epidermolysis bullosa simplex, Generalized pairwise comparison (GPC), Guidance, Model averaging, NparLD, GEE-like semiparametric model, Rare Diseases, Recommendation, Repeated measures, Small sample size

## Abstract

**Background:**

Recommendations for statistical methods in rare disease trials are scarce, especially for cross-over designs. As a result various state-of-the-art methodologies were compared as neutrally as possible using an illustrative data set from epidermolysis bullosa research to build recommendations for count, binary, and ordinal outcome variables. For this purpose, parametric (model averaging), semiparametric (generalized estimating equations type [GEE-like]) and nonparametric (generalized pairwise comparisons [GPC] and a marginal model implemented in the R package nparLD) methods were chosen by an international consortium of statisticians.

**Results:**

It was found that there is no uniformly best method for the aforementioned types of outcome variables, but in particular situations, there are methods that perform better than others. Especially if maximizing power is the primary goal, the prioritized unmatched GPC method was able to achieve particularly good results, besides being appropriate for prioritizing clinically relevant time points. Model averaging led to favorable results in some scenarios especially within the binary outcome setting and, like the GEE-like semiparametric method, also allows for considering period and carry-over effects properly. Inference based on the nonparametric marginal model was able to achieve high power, especially in the ordinal outcome scenario, despite small sample sizes due to separate testing of treatment periods, and is suitable when longitudinal and interaction effects have to be considered.

**Conclusion:**

Overall, a balance has to be found between achieving high power, accounting for cross-over, period, or carry-over effects, and prioritizing clinically relevant time points.

## Introduction

In the European Union, a rare disease is defined as one that affects less than 1 in 2000 people. Despite the low prevalence of a particular rare disease, the fact that there are over 6,000 rare diseases known to date makes them a huge challenge for healthcare systems, research, and—most importantly—the affected patients and families [10, 27].

Just like in any other area of medical research, the key conclusions of scientific publications are frequently based on quantitative data. Therefore, using sound statistical methods for analyzing these data is crucial for ensuring the validity of the conclusions drawn regarding diagnosis, prognosis, and treatment of patients. In research on rare diseases, there are some additional challenges that have to be adequately addressed, such as, for example, the heterogeneity of the disease, small sample sizes, limited data for study planning, difficulties in defining appropriate outcome measures, and above all a considerable trial burden for the patients [30].

The main aim of the present paper is to provide recommendations and guidance on statistical methods for analyzing longitudinally collected data in a cross-over trial setting. Considering the fact that sample sizes are usually small in rare diseases trials, this is a quite natural design choice. The basis for our recommendations is constituted by systematic empirical comparisons of existing methods that have been conducted in the context of the EBStatMax project (European Joint Programme on Rare Diseases, EU Horizon 2020 grant no. 825575). In this collaborative project, recently proposed methods for the statistical analysis of rare disease data have been extensively evaluated, motivated by longitudinal cross-over data from a clinical trial on Epidermolysis bullosa (EB). In the present manuscript, the findings from these extensive evaluations and related considerations are distilled into statistical guidance regarding design and analysis of such trials. It should be emphasized that the focus of this guidance is on rare disease trials featuring longitudinal and cross-over aspects. Some generally applicable lessons learnt from previous projects have partially informed the decision which data analysis methods should be considered. Yet, those further aspects of rare diseases methodology have been addressed in more detail elsewhere: In the ASTERIX [2], InSPiRe [20], and IDeAl [19] projects, a wide range of design and data analysis issues has been tackled, from surrogate endpoints (e.g., Elst et al. [8]) to adaptive designs (e.g., Graf et al. [15]) and pharmacometrics (e.g., Strömberg and Hooker [31]). In addition to numerous publications in statistical journals, the corresponding project consortia also released several summaries and guidance documents (*e.g.,* Hilgers et al. [17]).

In Sect. “Motivation”, we briefly review the motivating example of Epidermolysis Bullosa (EB), which forms the basis for both this guidance paper and the EBStatMax project. Reference is also made to the outcomes analyzed. Indeed, for a rare disease trial, different types of outcomes (“objective”, “patient-centered”, etc.) have to be considered. Statistically, these are measured on different scales, which is the key reason for presenting them separately in the sequel. After describing the simulation setup and the primary performance measures in Sect. “Simulation design and performance measures”, the used state-of-the-art methods for rare disease cross-over trials will be theoretically presented in Sect. “Methods”. More application-oriented readers may also skip this theoretical part, because it is not necessary to understand all technical details of the respective methods in order to comprehend the key results. Following this description of the methods, Sect. “Results and recommendations” presents the results and recommendations for the three outcomes considered in separate subsections. Finally, Sect. “Discussion” contains a discussion of the key results and summarizes the main conclusions. This manuscript is primarily intended to provide recommendations for applied researchers. At the same time, since the recommendations are based on systematic empirical comparative evaluations of statistical methods, it might also be of interest for statisticians and methodologists. Moreover, other stakeholders in the field of rare diseases, such as regulators, pharmaceutical companies, and above all, the patients, might also find these recommendations useful, due to the fact that choosing valid statistical methods is a key step in providing trustworthy evidence of efficacy of (novel) treatments.

## Scope of the guidance

### Motivation

**Fig. 1:**
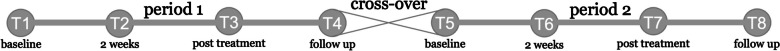
Illustration of the cross-over study design of the EB trial

Because of the inherent rarity of the disease, small sample sizes are frequently encountered in rare disease trials. This also applies to the inherited skin disease Epidermolysis Bullosa (EB), clinically characterized by fragility of epithelial-lined tissues and surfaces with recurrent mucocutaneous blistering. Treatment approaches try to ameliorate, among others, the blisters formation and their accompanying symptoms such as burdensome pain and pruritus. The starting point for these recommendations within the EBStatMax project is a data set from this area of research. The data set derives from a study in which EB patients were treated with an immunomodulatory topical diacerein cream. In a randomized, placebo-controlled, 2-period cross-over phase 2/3 trial, the impact of 1% diacerein cream vs. placebo in reducing the number of blisters in the simplex subtype of EB (EBS) was assessed. Furthermore, the severity of pain and pruritus were assessed by a visual analogue scale (VAS). The VAS ranges from 0 (no pain/pruritus) to 10 (worst pain/pruritus imaginable). Fifteen patients were randomized to either placebo or diacerein for a 4-week treatment and a 3-month follow-up period. After a washout phase, patients were crossed over to the opposite treatment for a second treatment period (see Fig. 1).

86% of the patients receiving diacerein, in period 1 and 37.5% in episode 2 met the primary endpoint, i.e., a reduction of the number of blisters by more than 40% from baseline in predefined assessment areas after the 4-week treatment (vs. 14% and 17% with placebo, respectively). Additionally, the blister counts as well as the VAS scores for pain and pruritus were considered as secondary outcome variables (see [35] for more details). Since these outcomes are measured on different scales (binary for the primary outcome and metric/count as well as ordinal for the secondary outcomes), recommendations for these three types of outcomes will be derived from previously conducted simulation studies [14, 33]. Moreover, in each of these two methodological papers, a real life data example was also described, using the original study data of Wally et al.[35]. It should be noted that other study designs for rare disease settings are both viable and common, but this guidance paper focuses on longitudinal studies and cross-over aspects.

### Simulation design and performance measures

EBStatMax is a so-called demonstration project funded by the European Joint Programme on Rare Diseases (EJP-RD), aimed at bridging the gap between challenges arising from clinical practice and their potential statistical solutions. To this end, at first, the members of the EBStatMax project consortium set up simulation studies, to compare different statistical methodologies. Subsequently, the project group addressed the key goal of the project, that is, to not only consider the methods theoretically but to also provide recommendations, software implementations, and educational materials. The aim of the simulations was to neutrally compare different statistical hypothesis testing approaches and to recommend methods that maintain the nominal type-I error while demonstrating competitive statistical power compared to other methods. We will describe the simulation scenario for ordinal outcomes (VAS scores for pain and pruritus) in detail here. The simulation scenarios are similar for count and binary outcomes. For the count outcome the raw blister counts were used. Finally, the binary outcome was created based on an indicator for a 40% reduction compared to baseline (for details regarding the respective simulation setup, see Geroldinger et al. [14] and Verbeeck et al. [33]).

**Fig. 2:**
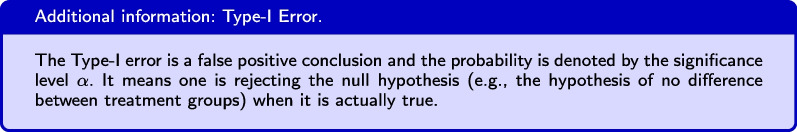
Type-1-error

The EB trial data set was used as the basis for the simulations. Recall from Sect. “Motivation” that this is a longitudinal data set from a cross-over study with 4 time points per subject and study period. In the simulations, the outcome measurements were grouped into blocks of four time points rather than considering individual time point levels. For each simulation run, the blocks per subject and treatment period were randomly permuted across all subjects and treatment conditions (placebo and verum). Permuting full blocks ensured that data characteristics (especially the longitudinal dependence structure within each block) was preserved. On average, over all simulated samples, the block permutation established a situation of no difference between the treatment conditions and, hence, allows to evaluate the empirical type-I error rates (for more details regarding the type-I error please see the Fig. 2 in Sect. “Discussion”).

**Fig. 3:**
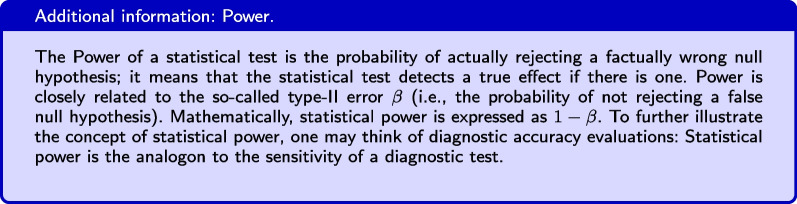
Power

For power simulations, additional steps were implemented to simulate different treatment effect scenarios. These steps included generating random variables from different distributions and adding them to the observations from the placebo group at specific time points (because larger VAS scores / blister counts are considered as worse outcomes). The setup for power simulations aligns with clinical expertise by considering different distributions (for more details regarding power in general, please see the Fig. 3 in Sect. “Discussion” and see Geroldinger et al. [14] and Verbeeck et al. [33]). The parameters of these distributions were chosen such that the expected values (shift effects) corresponded to clinically meaningful effects. Since the outcomes are observed longitudinally, that is, at 4 time points within each period, the time point(s) when the effect was present had to be specified. To this end, two different scenarios were considered:

- *Scenario 1* The random variables were added under placebo at the third time point (i.e., the post-treatment visit) only (=scenario with a single time point with a treatment effect).
- *Scenario 2* The random variables were added under placebo at the third time point (i.e., the post-treatment visit), and additionally, about half of the effect was added to outcome under placebo at the fourth time point, *i.e.,* the follow-up visit (=scenario with multiple time points with a treatment effect).

The resulting empirical power values were based on a two-sided level of significance ($\alpha = 0.05$).

### Methods

In this section, different approaches are presented for analyzing count, binary, and ordinal outcome measures in rare disease settings, respectively, if a cross-over study is conducted. Based on these methods, recommendations are then suggested. At the outset, however, it is important to distinguish between three different basic approaches. Indeed, there are parametric, semiparametric and nonparametric approaches that can be used. A large class of statistical methods is of a parametric nature; this means that a distribution is assumed for the data, and in particular, for longitudinal settings a correlation structure of the measurements within a subject is fully specified. Differences between two groups, for example, are then quantified by differences or ratios of certain parameters, such as means, medians, or other regression parameters. Results that emerge from these parametric procedures usually depend substantially on the extent to which the observed data in the sample can be modeled by these parametric distributions, i.e., how well the model fits the data, and how sensitive the chosen methods are against violations of the parametric assumptions. Nonparametric statistics is a key counterpart to this, not requiring the use of specific distributional assumptions at all. Thus, nonparametric approaches can be used to analyze metric, ordinal, categorical, or binary variables, either discrete or continuous, while a parametric approach would require distinct models for each of these variable types. Semiparametric methods, in contrast, have both parametric and nonparametric features; for example, one might assume a parametric mean-based model, while the correlation structure of the measurements is not fully specified [4, 5].

In the sequel, different parametric, semiparametric and nonparametric approaches are discussed, which have been selected as competitors for the simulation studies (see Sect. “Simulation design and performance measures”). The choice of these approaches has been made by the project consortium and guided by extensive previous experience with applying these methods in the context of small samples / rare diseases. For a more detailed theoretical description of these methods, we refer to [14, 33], and the references therein.

#### Nonparametric marginal model (NMM)—nparLD

A nonparametric marginal model (NMM) is implemented in the R package nparLD. This provides easy and user-friendly access to robust rank-based methods for the analysis of longitudinal data in factorial settings. For model classification purposes, nparLD uses a notation system for frequently used factorial designs depending on the number of factors. To this end, the factor which stratifies samples into independent groups, is called a *whole-plot factor*, while the factor defining the repeated measurements is of a so-called *sub-plot-factor* [28] type. This terminology derives, historically, from agricultural experimental design. The underlying effect size for this method is the so-called relative effect. To illustrate the simplest case for the relative effect mathematically, the version for two random variables is defined as follows [4]. For two independent random variables $X_1 \sim F_1$ and $X_2 \sim F_2$, the probability

1 $$\begin{aligned} p = P(X_1 < X_2) + \frac{1}{2} P(X_1 = X_2) \end{aligned}$$

is called *relative effect* of $X_2$ with respect to $X_1$ [4, 5]. This definition is based on the observation that for two independent continuous random variables $X_1$ and $X_2$, the relative effect of $X_2$ to $X_1$ can be characterized by

$$p^+ = P(X_1 \le X_2) = P(X_1 < X_2) = 1- P(X_1 \ge X_2),$$

i.e., the probability that $X_1$ takes smaller values than $X_2$, since in the continuous case $P(X_1 = X_2) = 0$. Informally speaking, the relative effect quantifies the probability that the values of the outcome of one group are smaller than in the other group. Moreover, it immediately follows from its definition that the relative effect *p* takes values between 0 and 1.

To perform appropriate test procedures using the R package nparLD, the relative effect is used to derive test statistics. For the purpose of the EBStatMax project, that is, for the analysis of the time course within a cross-over study, it was decided to test for an interaction effect within this framework, i.e., to test whether the longitudinal profiles are different between the two treatments. To this end, the so-called *ANOVA type statistic* (ATS) was used, which can be regarded as a nonparametric generalization of the classical parametric ANOVA test statistic. In particular, its sampling distribution can be approximated by an *F* distribution (for details, see [4, 28]).

#### Generalized pairwise comparisons (GPC) variants

Another nonparametric approach is the so-called Generalized Pairwise Comparisons (GPC). If there is only a single outcome and no missing data, the GPC approach is a linear transformation of the well-known Mann-Whitney rank test [25, 32]. Instead of a rank-based approach, the GPC method evaluates the longitudinally measured outcomes by constructing all possible pairs, one from each treatment group, and subsequently assigns a score to each pair. Since pairs are constructed between the two treatment arms independently from the treatment period, period effects are ignored in this method.

Intuitively, there are different approaches to construct these pairs for longitudinally collected outcomes. In mathematical terms, one could choose a univariate or multivariate approach, distinguish between matched and unmatched versions, and prioritized or non-prioritized methods. In each case, a score, denoted by $U_{k\ell }$, is assigned, corresponding to the comparison of the univariate or multivariate outcome, denoted by $V_{1k}$, for patient *k* under verum and $V_{2\ell }$ for patient $\ell$ under placebo, as follows:

2 $$\begin{aligned} U_{k\ell }=\left\{ \begin{array}{rl} 1, &{}\text {if } V_{1k}>V_{2\ell } \\ -1, &{}\text {if } V_{1k}<V_{2\ell } \\ 0, &{}\text {if } V_{1k}=V_{2\ell }, \end{array} \right. \end{aligned}$$

In a univariate approach, the repeated measurements are evaluated by constructing one summary measure per subject and per treatment period and by comparing these summary measures within a pair. On the other hand, a multivariate approach allows for the evaluation of the longitudinal outcomes by comparing the outcomes per time point *t*, either ordered in a prioritized way or not ordered (non-prioritized). In a prioritized approach for repeated measurements, the time points are prioritized by importance. For each pair, a score is assigned by applying the rule defined in (2) to the first-ranked time point. If and only if the score results in a tie, the next ranked time point is evaluated in the pair, continuing until the last time point [6, 13, 29, 34]. Furthermore, it is possible to consider the treatment periods of a subject as independent, which ignores the cross-over effect and is called an unmatched approach, leading to asymptotically valid results. Alternatively, pairs can be restricted to compare treatment periods only within subjects, in a matched approach.

After choosing the desired variant and assigning a score, various statistics can be constructed. An easy to interpret and frequently used treatment effect size, is the so-called net treatment benefit ($\Delta$). For example, the unmatched net treatment benefit is defined as the sum of scores divided by the total number of pairs:

$$\begin{aligned} \Delta _{unm}= \frac{1}{n^2}\sum _{k=1}^{n}\sum _{\ell =1}^{n}U_{k\ell }. \end{aligned}$$

The range is $[-1,+1]$, which is easy to interpret as the difference of probability that a random subject will do better on active treatment than on placebo and vice versa.

Various testing procedures are available for the GPC variants. Due to the small sample sizes, permutation tests based on the null hypothesis are used for unmatched approaches:

$$\begin{aligned} H_0: \bar{F}_{1.}= \bar{F}_{2.}, \end{aligned}$$

with $\bar{F}_{i.}$ defining the distribution of the outcome in treatment group *i*. For matched variants, the conditional sign test is used to take ties and also the small sample size into account (see Coakley and Heise [9]; Dixon and Massey [7]; Fagerland [36]; Wittkowski [12] for more details).

#### GEE-like semiparametric model

Modelling fully the two types of covariance patterns present in repeated-measures cross-over designs—within-treatment period dependencies and between-treatment period dependencies—may be challenging in small samples. Generalized estimating equations (GEE) and, more generally, semiparametric methods, including those based on pseudo-likelihood, avoid the need for full likelihood specification for Gaussian and non-Gaussian data [23]. GEE-like models involves, for example, specifying pairwise densities for repeated measures, which substitute the full likelihood with the product of each possible pair of measures [26]. A variety of other versions exists. Bias-corrected sandwich estimators are available for small sample sizes, which lead to consistent estimators when correctly specified [24]. Assuming independence of the between- and within-period covariance structures, the between-period dependencies are modelled through fixed subject effects, while the within-period dependencies are modelled using a residual covariance structure [21]. Considering $i = 1, 2$ for the treatment assignment, $k = 1,\dots , N$ for the subjects and $t = 1,\dots , 4$ for the time points, the blister count $X_{ikt} \sim Poisson(\lambda _{ikt})$ and the dichotomized blister outcome $Y_{ikt} \sim Bernoulli(\pi _{ikt})$ are modelled by:

3 $$\begin{aligned} \text{ logit }(\theta _{ikt})= \beta _0 + \beta _1 G_{ik} + \beta _2 P_k + \sum _{j=3}^5 \beta _j T_{ijkt} + \beta _6 G_{ik}P_k + \sum _{j=7}^9 \beta _j G_{ik}T_{ijkt} + \sum _{j=10}^{12} \beta _j P_kT_{ijkt}, \end{aligned}$$

where $\theta _{ikt}$ is either $\lambda _{ikt}$ or $\pi _{ikt}$, and with $G_{ik}$ a treatment group indicator, $P_k$ a period indicator and $T_{ijkt}$ a discrete time indicator. In these models, a Wald test for an overall treatment effect can be used, by evaluating a linear combination of parameters involving the treatment group indicator $L\beta = 0$. The treatment effect is expressed as the odds ratio for the dichotomized blister outcome and the rate ratio for the count outcome. The SAS procedure GLIMMIX supports repeated-measures designs, including cross-over designs, and hence can accommodate carry-over effect evaluation, while accommodating missing data. The results are valid when data is missing at random, provided likelihood-based estimation is used. When a non-likelihood estimation method is used, it is advisable to pre-process the data using multiple imputation.

#### Model averaging (MA)

It is often seen that, for the analysis of longitudinal data, parametric models are the most powerful methods for hypothesis testing [22]. However, the methods require models to fully specify the time changing aspects of the data as well as the variance across individuals (at a minimum, for discrete data). For studies with small samples and relatively little previous information about the data structure, finding an optimal model for the system may be challenging [3, 18]. One way to circumvent these limitations in small samples is to pre-define a range of models and to use a weighted average of metrics of interest across all models [1]. This approach is sometimes also called ensemble modeling.

In model averaging (MA), the weighting of the models is typically done according to their fit to the data (AIC, for example), but other weight factors can be used. These approaches are commonly applied in, for example, weather forecasts, where numerous models are used to make predictions of the coming weather. Those predictions are then weighted to give one single prediction, including the uncertainty of that prediction, based on model fits to available weather data.

A standard model averaging procedure to identify treatment effects in a clinical trial would involve:

- Predefining models that fit to the data
- Determining the weights for each of the models according to model fit to data. For example, the weight $w_q$ for the $\mathcal {M}_q$ model in the pool of candidate models $\textbf{M}$ ($\textbf{M}=\{\mathcal {M}_1,\ldots ,\mathcal {M}_Q\}$) models could be determined according to
  4 $$\begin{aligned} w_q=\frac{e^{-\frac{1}{2} \text {AIC}q}}{\sum _{q'=1}^Q e^{-\frac{1}{2}\text {AIC}_{q'}}} \end{aligned}$$
- Computing effect measures and uncertainty of those effect measures using a weighted prediction from the candidate model set.

## Results and recommendations

### Count outcomes

For count data, the number of blisters from the original study of Wally et al. [35] was used as the outcome variable. For the analysis, the parametric, semiparametric, and nonparametric methods that have been introduced in Sects. “Nonparametric marginal model (NMM)—nparLD”, “Generalized pairwise comparisons (GPC) variants”, “GEE-like semiparametric model” and “Model averaging (MA)”– were compared, namely NMM (nparLD), GPC Variants, GEE-like models, and model averaging.

Firstly, it was found that the type-I error is controlled by almost all methods except for model averaging and matched GPC approaches. For the matched GPC models this is due to the fact that the number of pairs is too small, as the conditional sign test requires at least 15 matched subjects (see Fagerland et al. [11] or Coakley and Heise [7] for more details), which were not available within this data set.

It is important to note that the underlying null hypotheses for the compared methods are different. For example, the null hypotheses accompanying the nonparametric methods are more restrictive compared to GEE-type and model averaging approaches. So, although an attempt was made to take a neutral position for deriving the recommendations, the fact that the underlying hypotheses are different must be kept in mind when interpreting the results provided in what follows.

Multivariate GPC methods (prioritized and non-prioritized) resulted in the highest power values to detect a treatment effect. Prioritized GPC leads to the highest power for scenarios with a single time point with a treatment effect (i.e., Scenario 1 in Sect. “Simulation design and performance measures”), followed by model averaging. Non-prioritized GPC yields the highest power for scenarios with multiple time points with a treatment effect (i.e., Scenario 2 in Sect. “Simulation design and performance measures”), closely followed by unmatched prioritized GPC. It should be noted that although prioritization was based on clinical grounds, it may have led to some bias towards favoring the method by design (see Verbeeck et al. [33] for more details). Matched GPC approaches resulted in lower power values than unmatched counterparts. It should be noted that work by Fagerland et al. [11] suggests that matched GPC approaches only lead to meaningful results with a sample size of about $N>15$. Within-period and within-subject dependence modeling in a cross-over trials using a GEE-type model do not produce a distinct advantage over nonparametric methods. The power is comparable to matched GPC and lower than unmatched GPC; also, there may be convergence problems. Parametric model averaging approaches are computer intensive and do not lead to higher power compared to nonparametric GPC variants, but on the other hand, similarly as the GEE-like semiparametric model, they allow for evaluating if a carry-over or a period effect is present.

The nonparametric marginal model (NMM), which is implemented in the R package nparLD, can be used for testing the interaction effect between treatment and time. Hence, this means that NMM is suitable for detecting differences between groups regarding the longitudinal profiles. At this point it would have been interesting to compare GEE-type models and model averaging models with NMM, since these approaches can also test interaction effects. However, since such sort of comparative simulations have not been carried out, no recommendations for evaluating differences between profile lines are provided. However, the results of the simulations that have been carried out for NMM are briefly summarized as follows. The simulations have shown that the nonparametric marginal model (NMM) is good at detecting a profile with a peak (i.e., a single time point with a treatment effect), yet much less so for a longitudinal profile with a treatment effect on multiple time points. Additionally, the nonparametric marginal model requires to test each treatment period separately and thus does not allow for considering a cross-over effect.

**Fig. 4:**
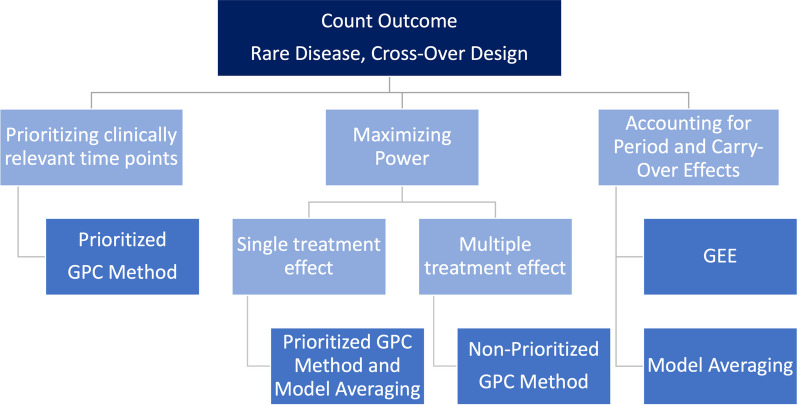
Recommendations for count outcomes in cross-over rare disease trials

### Binary outcomes

The primary endpoint of the study conducted by Wally et al. [35] was the proportion of patients with more than 40% reduction from baseline in the number of blisters after 4 weeks of treatment. This binary outcome was also chosen as the starting point for the simulations. Overall, we evaluated the same methods as for the count outcome (see Sect. “Count outcomes”) in order to make a thorough empirical comparison in terms of the resulting type-I error and power. The methods under consideration were NMM, GPC variants, GEE-type, and model averaging. Dichotomizing count data, as given by the number of blisters, leads to loss of granularity in the data. This translates in our results to lower power values to detect a treatment effect in the dichotomized blister counts compared to the raw blister counts. Therefore, most tested methodologies are more sensitive to detect a treatment effect using a count outcome compared to the usage of a binary outcome, as provided by dichotomization of blister counts (see [33] for more details).

NMM is a liberal testing procedure, and has higher power values when there is only one treatment visit with a treatment effect. Furthermore, it is limited by the fact that there is only two-sided testing possible. GPC controls type-I error well, except for the non-prioritized unmatched version, possibly due to a high amount of ties due to dichotomization. Similarly to the count outcome, the unmatched versions lead to higher power values than the matched counterparts (see Sect. “Count outcomes” for more details).

For GEE-type semiparametric models, small-sample corrections are not necessary, given that the variance for a dichotomized count is closer to homoscedasticity compared to count data. The power is close to the matched GPC, but lower than the unmatched GPC. For model averaging, the difference between treatment and reference groups in terms of the proportions of the dichotomized outcome was used, which is commonly referred to as a $\Delta \Delta$ effect measure. The type I error is controlled; with respect to power, MA outperforms the other competitors in scenarios with a single time point with a treatment effect, while it is comparable to GPC in scenarios with multiple treatment effects.

**Fig. 5:**
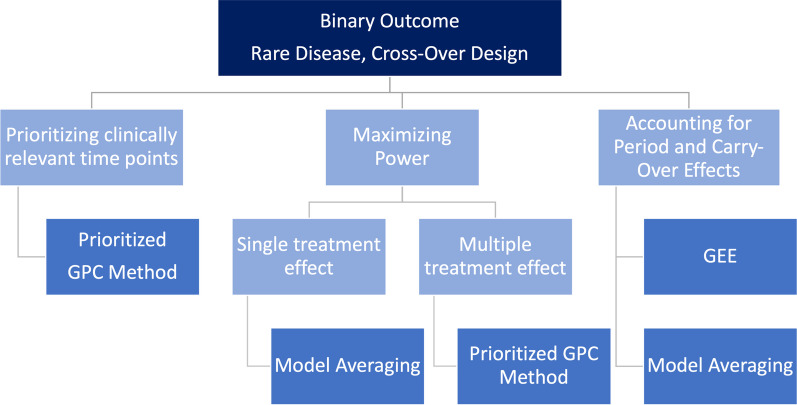
Recommendations for binary outcomes in cross-over rare disease trials

### Ordinal outcomes

For ordinal outcomes, the nonparametric rank-based marginal model (NMM) using the R package nparLD was compared with various generalized pairwise comparisons (GPC) methods. Especially for ordinal outcomes, only these two approaches were included in the comparison, since they are based on nonparametric approaches and are therefore very suitable for this type of outcome.

For the selected context of the EB data set, the ordinal outcomes were given by the visual analogue scale (VAS). This score was used for the outcomes “pain” and “pruritus” and ranged from 0 (no pain/pruritus) to 10 (worst pain/pruritus imaginable) with an increment of 0.5 [35]. Note, that the visual analogue scale usually consists of a line of 100 mm in length, with anchor descriptors such as “no pain/pruritus” and “worst pain/pruritus imaginable” where patients indicate their level of pain or pruritus. Importantly, VAS scores are considered as being measured on an ordinal scale since differences cannot be interpreted in an uniform, meaningful way. For example, in clinical practice, a decrease in VAS from 8 to 6 might be interpreted differently than a decrease from 3 to 1 [16]. We have already outlined the basic concept of the two methods NMM and GPC in more detail in Sects. “Nonparametric marginal model (NMM)—nparLD” and “Generalized pairwise comparisons (GPC) Variants”, respectively.

For the aforementioned study design of a longitudinal cross-over study, we attempted to select statistical methods that could reasonably account for this study type. However, it must be noted that the NMM method implemented in nparLD can only handle the periods separately at this stage. Thus, each treatment period is considered separately and the interaction effect of the considered outcome of the VAS score is calculated by a rank-based test statistic for each of the two periods separately. All evaluated methods control the type-I error, except for the matched GPC variant. Among all tested methods, the prioritized GPC variant achieved the highest power. The name of the method indicates, however, that specifying a clinically relevant and meaningful prioritization is a key requirement for using this method. Since for NMM, periods had to be evaluated separately, the sample sizes were particularly low (i.e., a sample size of $N=6$ patients). However, NMM could still achieve very high power values, suggesting that this method can be recommended also for very small sample sizes. The longitudinal structure can be analyzed properly with both unmatched GPC variants and NMM. Furthermore, the NMM is also capable of detecting interaction effects (i.e., detecting significant differences between longitudinal profiles). As a conclusion, the results of Geroldinger et al. [14] indicate that especially for longitudinal data in a small sample size cross-over study, we can derive some recommendations indeed. At the same time, however, perhaps even more than in the binary and count outcome cases, a trade-off must be made between increasing power and analyzing period-specific effects.

**Fig. 6:**
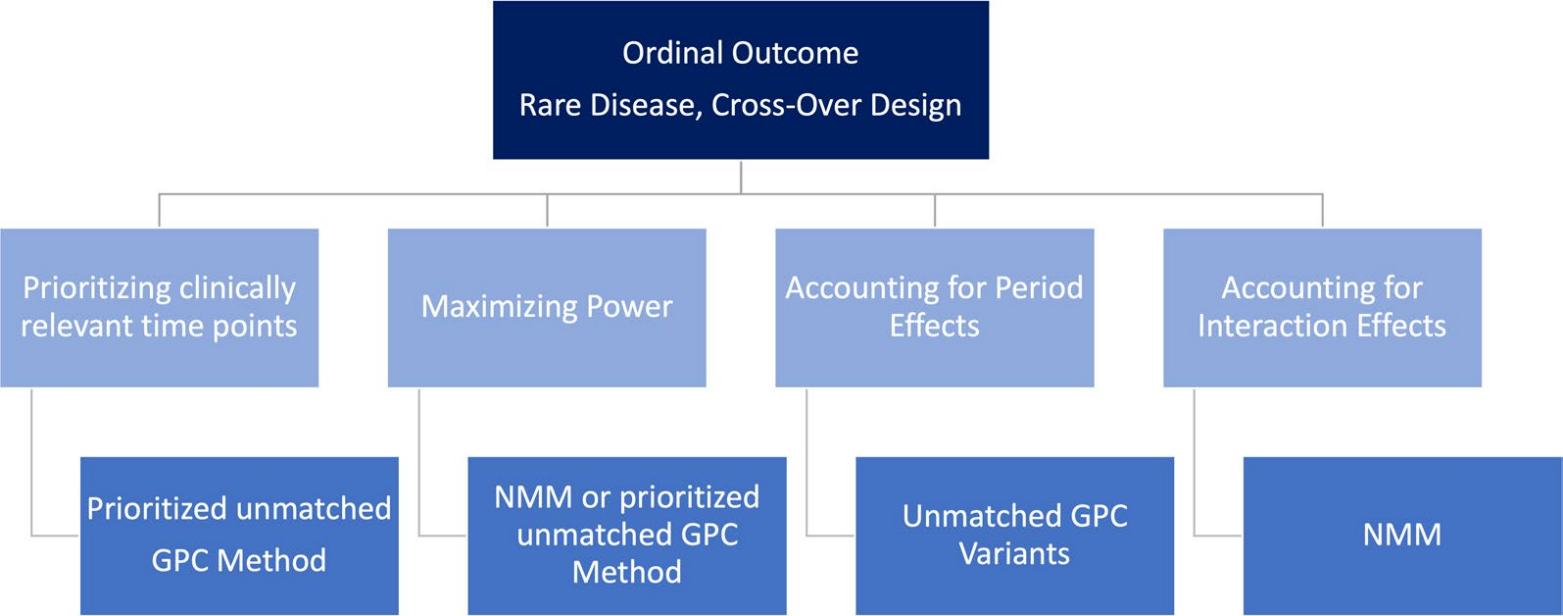
Recommendations for ordinal outcomes in cross-over rare disease trials

### Real-life data application

The given results are based on several simulation studies, which were performed in the works of Geroldinger et al. [14] and Verbeeck et al. [33], also using real-life data examples. Instead of just duplicating the corresponding subsections of those papers, the present manuscript provides an application-oriented summary of the results and derives recommendations for practice. Still, however, it should be emphasized that these recommendations regarding the three analyzed outcomes (count, binary, and ordinal) are based on the original data set of the study by Wally et al. [35]. In the sequel, we therefore briefly illustrate the application of our recommendations (Figs. 4, 5 and 6) to this concrete data example: The primary focus in the Diacerein trial conducted by Wally et al. [35] was to use a statistical method that maximizes power, since the recruitment of study subjects in Epidermolysis Bullosa—and in rare diseases in general—is usually quite difficult. Therefore, the sample size is very small. In addition, the time point immediately after the end of treatment is the clinically most relevant one, assuming that there is the peak of the therapeutic effect. Yet, also sustained long-term effects of the treatment are of interest, and therefore, the last time point (i.e., at the end of follow-up) is relevant, too. Thus, a prioritization of time points would be desirable. By contrast, accounting for period-, interaction, and/or carry-over effects is somewhat less important, since some measures to avoid such effects have been taken already at the design stage. Thus, as an example, we will now demonstrate how to use our recommendations to identify an appropriate analysis method for ordinal outcomes: In the real-life data set of Wally et al. [35], ordinally scaled outcomes were pain, pruritus, and quality of life. According to Fig. 6, this would mean that we could use the prioritized unmatched GPC variant due to the desired prioritization, which would also be recommended for the maximization of the power. Analogously, due to the given clinical focus, we would obtain the respective methodological recommendations for the count and binary outcome by the respective figures (Figs. 4 and  5) as well.

Finally we would like to emphasize once more that in other real-life data applications one might put more emphasis on period- or carry-over effects, and depending on this decision, different recommendations and methods may be applied. In summary, this implies that one can refer to these given recommendations in case of a longitudinal study design with cross-over. Any number of measurement time points within the respective treatment periods can be dealt with in this case. When applying these statistical recommendations, it is important to consider possible clinical aspects that appear to be crucial for the real-life data application. These may include whether it would be beneficial to prioritize study time points, to give greater consideration to carry-over or period effects, or to emphasize the importance of maximizing the statistical power of the applied method. Indeed, depending on this decision, different methods are recommended, as shown in the respective figures (Figs. 4, 5 and 6) for the different scales of the outcome variables.

## Discussion

The aim of this work was to provide some practically oriented guidance regarding the use of different statistical methods for small sample sizes in a cross-over trial. For this purpose, the starting point was a study by Wally et al. [35], in which data on count, binary and ordinal outcomes was collected. Previously, neutral simulation studies that are based on this data set have been performed and published (see Geroldinger et al. [14] and Verbeeck et al. [33] for more details, in particular for real-life data examples). To provide a more condensed summary of the recommendations that can be derived from these rather technical publications, we provide this guidance document regarding the analysis of trials with a cross-over design with repeated measures. It should be mentioned that those recommendations are given on the basis of one single data set, and simulations based on permutations of this original data. Future work should be aimed at including also other data sets to further validate the given recommendations.

Overall, different statistical state-of-the-art methods were compared, including parametric, semiparametric, and nonparametric approaches. The methods were selected by an international consortium within the so-called EBStatMax project. We compared rank-based and nonparametric methods, namely the generalized pairwise comparison (GPC) variants (univariate, multivariate, matched, unmatched, prioritized and non-prioritized) and inference based on a nonparametric marginal model (NMM, as implemented in the R package nparLD), the semiparametric GEE-like model and parametric model averaging approaches. Overall, for all three tested levels of measurement (count, binary and ordinal), it could be demonstrated that the power and the results strongly depend on the level of measurement of the outcome.

Based on our simulations, especially the prioritized unmatched GPC method was able to achieve particularly high power values and could lead to good results in many scenarios. This multivariate approach allows to analyze count, binary and ordinal outcomes and to prioritize them by time point. For each pair, a score is assigned to the first ranked time point. If and only if the score results in a tie, the next ranked time point is evaluated, continuing until the last time point. The prioritization of the time points therefore has a big impact on power. This makes sense, since the prioritization that was specified in the GPC closely corresponds to the simulation settings (i.e., in the simulations, the effect was added in each scenario at the post treatment visit, which was, on the other hand, evaluated first in the prioritized analyses). However, we would like to emphasize that the reason for setting up the simulations and prioritizing with the GPC method in this way were clinical considerations and not the intention to favor this particular method. It should be emphasized that a different prioritization could also lead to inferior performance.

That being said, it is noteworthy that the NMM approach has a high power that is quite close to the prioritized GPC method, at least in some cases for example within ordinal outcome settings, even though the simulations were performed with a smaller sample size (group sizes of 6 and 7), which is due to the separate testing of the time periods. Future research could address this aspect and analyze parallel group designs in detail using these tested methodologies and to derive further recommendations for this study design. However, it should be noted that NMM as implemented in nparLD does not account for cross-over effects, because of the separate testing procedure of the treatment periods. Yet, NMM can account for the longitudinal profile line information and interaction effects. GEE-type models and model averaging might also be used to test interaction effects. Future work could link these to derive recommendations in this respect as well.

Furthermore, note that the dichotomization of count data leads to a loss of granularity in the data. It was found that all tested methods had less power in the binary setting than in the count data counterpart. But, model averaging led to similarly high power values as the prioritized unmatched GPC method, especially for the binary outcome, where other tested methods performed worse. Both MA and GEE-like models can take account of period or carry-over effects. Regarding the power of GEE and matched GPC approaches, however, it was found that they performed worse than comparative methods such as the prioritized unmatched GPC. For matched GPC approaches we are suggesting that it may not be appropriate for less than 15 subjects.

**Fig. 7:**
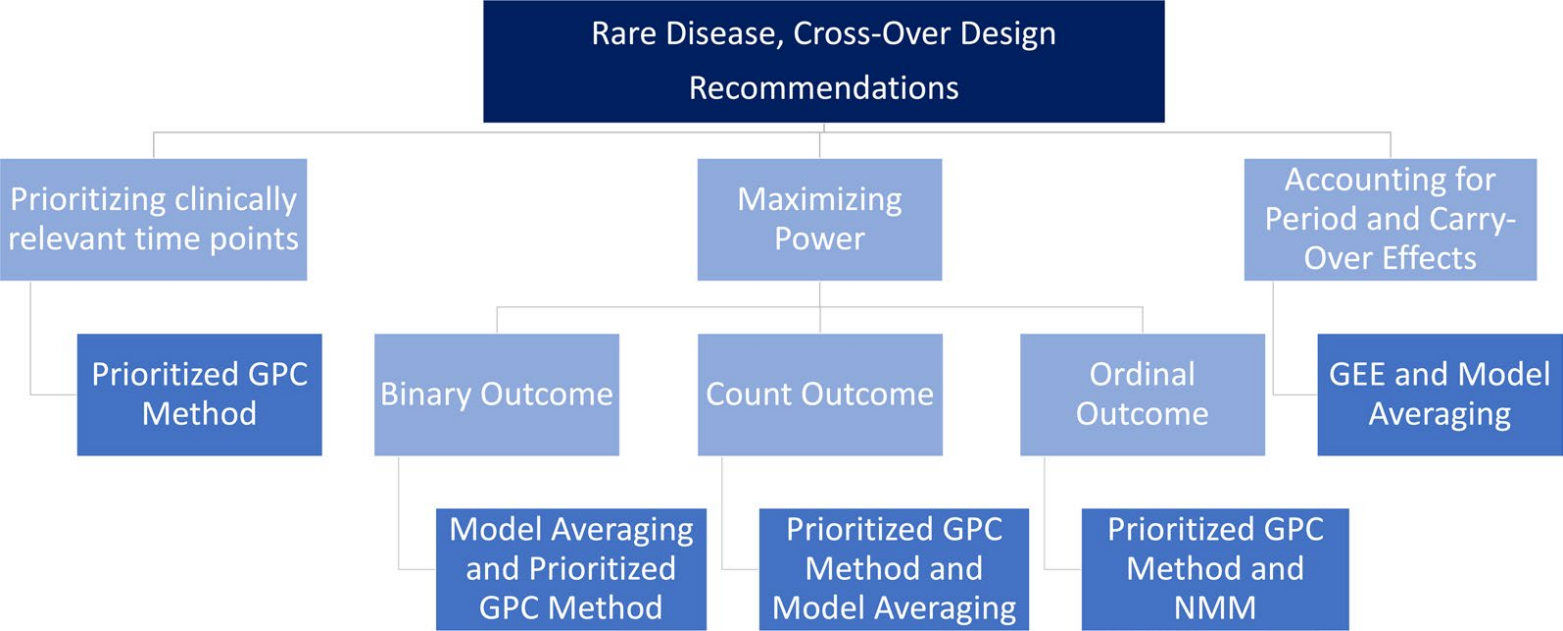
Overall recommendations for cross-over rare disease trials

In summary, our previous analyses, which constitute the basis for the recommendations provided in this paper, indicate that in certain scenarios some methods work better than others, yet there is no single method that outperforms the others in all scenarios. Therefore, Fig. 7 has no decision tree structure, but reflects a balance between different aspects (e.g., prioritizing relevant time points, or maximizing power). As an applied researcher being confronted with the task of choosing the appropriate statistical analysis method in such a rare disease cross-over setting, it must therefore be decided at the outset which priorities should be set for the statistical analysis. Altogether, Fig. 7 shows the overall recommendations in a simplified and combined way, thereby summarizing the outcome-specific recommendations and corresponding Figs. 4, 5 and 6.

It can be concluded that a balance between achieving high power, accounting for cross-over, period or carry-over effects, and prioritizing clinically relevant time points must be found.

## Data Availability

The data that support the findings of this study are available from the corresponding author upon reasonable request. The underlying original data and the simulation code is available in the following public repository in Github: https://github.com/martingeroldinger/Diacerein_study_data.
